# Supplementary material for: Altered microRNA Transcriptome in Cultured Human Liver Cells upon Infection with Ebola Virus
Source: Int J Mol Sci. 2021 Apr 6;22(7):3792. doi: 10.3390/ijms22073792 (PMC8038836; doi:10.3390/ijms22073792)
Supplement: Supplementary file 1 [file ijms-22-03792-s001.zip › Supplementary_File/C_ GO_Analysis_Results/16-30nt_go_Reston-96h-Huh7_vs_Control-96h-Huh7_down.mature_mirna_targets/CC_Pvalue_tree.pdf]

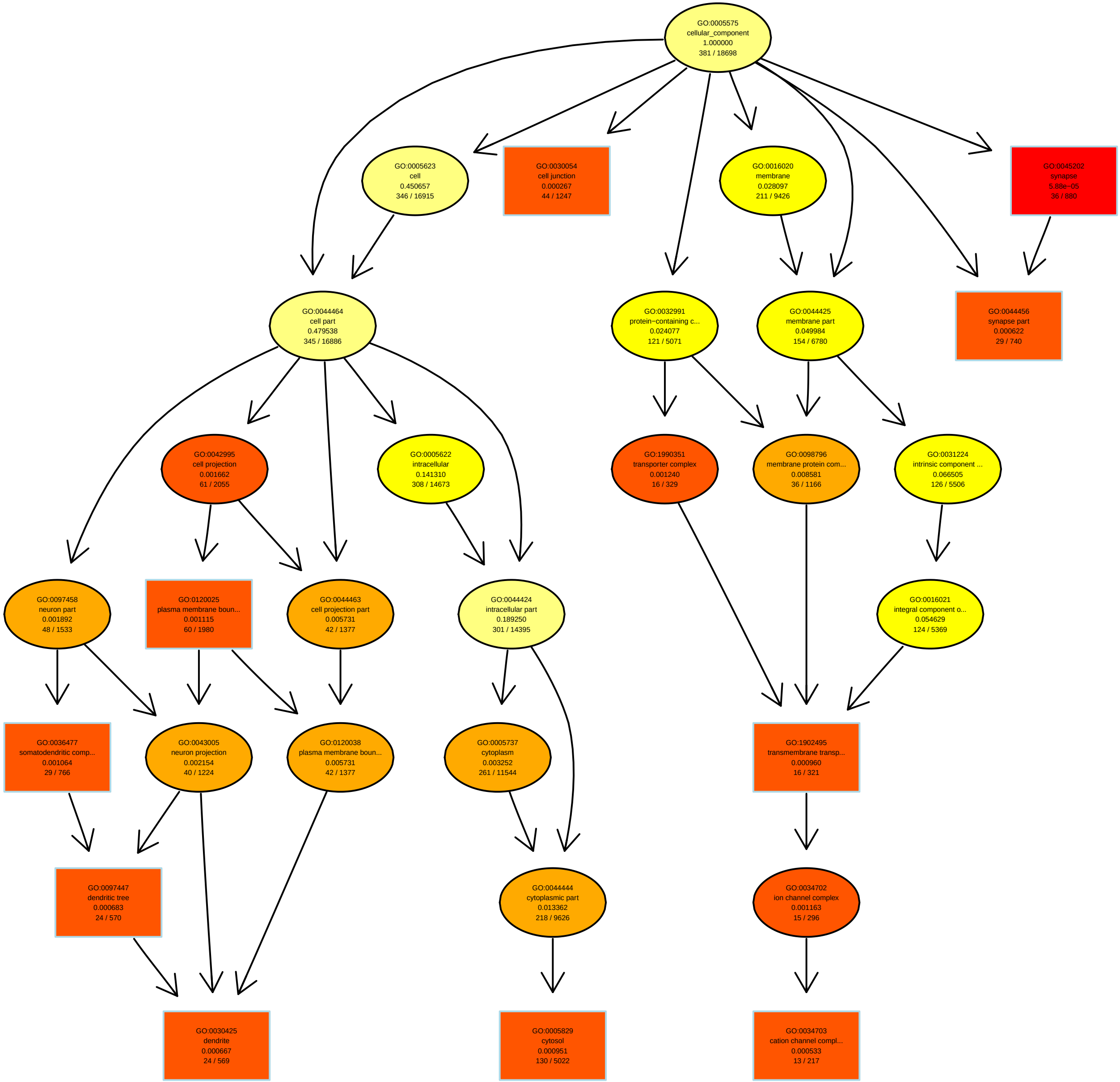

GO:0005575
cellular_component
1.000000
381 / 18698
GO:0005623
cell
0.450657
346 / 16915
GO:0030054
cell junction
0.000267
44 / 1247
GO:0016020
membrane
0.028097
211 / 9426
GO:0045202
synapse
5.88e−05
36 / 880
GO:0044464
cell part
0.479538
345 / 16886
GO:0032991
protein−containing c...
0.024077
121 / 5071
GO:0044425
membrane part
0.049984
154 / 6780
GO:0044456
synapse part
0.000622
29 / 740
GO:0042995
cell projection
0.001662
61 / 2055
GO:0005622
intracellular
0.141310
308 / 14673
GO:1990351
transporter complex
0.001240
16 / 329
GO:0098796
membrane protein com...
0.008581
36 / 1166
GO:0031224
intrinsic component ...
0.066505
126 / 5506
GO:0097458
neuron part
0.001892
48 / 1533
GO:0120025
plasma membrane boun...
0.001115
60 / 1980
GO:0044463
cell projection part
0.005731
42 / 1377
GO:0044424
intracellular part
0.189250
301 / 14395
GO:0016021
integral component o...
0.054629
124 / 5369
GO:0036477
somatodendritic comp...
0.001064
29 / 766
GO:0043005
neuron projection
0.002154
40 / 1224
GO:0120038
plasma membrane boun...
0.005731
42 / 1377
GO:0005737
cytoplasm
0.003252
261 / 11544
GO:1902495
transmembrane transp...
0.000960
16 / 321
GO:0097447
dendritic tree
0.000683
24 / 570
GO:0044444
cytoplasmic part
0.013362
218 / 9626
GO:0034702
ion channel complex
0.001163
15 / 296
GO:0030425
dendrite
0.000667
24 / 569
GO:0005829
cytosol
0.000951
130 / 5022
GO:0034703
cation channel compl...
0.000533
13 / 217
